# The Prognostic Role of the Immune Microenvironment in Sinonasal Intestinal-Type Adenocarcinoma: A Computer-Assisted Image Analysis of CD3^+^ and CD8^+^ Tumor-Infiltrating Lymphocytes

**DOI:** 10.3390/jpm13050726

**Published:** 2023-04-25

**Authors:** Marco Ferrari, Lara Alessandrini, Enrico Savietto, Diego Cazzador, Gloria Schiavo, Stefano Taboni, Andrea L. C. Carobbio, Leonardo Calvanese, Giacomo Contro, Piergiorgio Gaudioso, Enzo Emanuelli, Marta Sbaraglia, Elisabetta Zanoletti, Gino Marioni, Angelo P. Dei Tos, Piero Nicolai

**Affiliations:** 1 Section of Otorhinolaryngology—Head and Neck Surgery, Department of Neurosciences, “Azienda Ospedale Università di Padova”, University of Padua, 35128 Padua, Italy; diego.cazzador@aopd.veneto.it (D.C.); gloria.schiavo@aopd.veneto.it (G.S.); stefano.taboni@aopd.veneto.it (S.T.); andrea.carobbio@aopd.veneto.it (A.L.C.C.); leonardo.calvanese@gmail.com (L.C.); giacomo.contro@aopd.veneto.it (G.C.); piergiorgio.gaudioso@aopd.veneto.it (P.G.); gino.marioni@unipd.it (G.M.); piero.nicolai@unipd.it (P.N.); 2 Guided Therapeutics (GTx) Program International Scholarship, University Health Network (UHN), Toronto, ON M5G1L7, Canada; 3 Technology for Health (PhD Program), Department of Information Engineering, University of Brescia, 25123 Brescia, Italy; 4 Section of Pathology, Department of Medicine, “Azienda Ospedale Università di Padova”, University of Padua, 35128 Padua, Italy; lara.alessandrini@aopd.veneto.it (L.A.); marta.sbaraglia@unipd.it (M.S.); angelo.deitos@unipd.it (A.P.D.T.); 5 Unit of Otolaryngology, Hospital of Treviso AULSS 2-Marca Trevigiana, 31100 Treviso, Italy; enrico.savietto@aulss2.veneto.it (E.S.); enzo.emanuelli@aulss2.veneto.it (E.E.); 6 Artificial Intelligence in Medicine and Innovation in Clinical Research and Methodology, Department of Clinical and Experimental Sciences, University of Brescia, 25100 Brescia, Italy

**Keywords:** paranasal sinus neoplasms, adenocarcinoma, lymphocytes, tumor-infiltrating, prognosis, survival, natural orifice endoscopic surgery

## Abstract

The prognostic value of conventional histopathological parameters in the sinonasal intestinal-type adenocarcinoma (ITAC) has been debated and novel variables should be investigated. Increasing evidence demonstrated that the evolution of cancer is strongly dependent upon the complex interactions within tumor microenvironment. The aim of this retrospective study was to assess the features of immune microenvironment in terms of CD3^+^ and CD8^+^ cells in a series of ITAC and explore their prognostic role, as well as their relations with clinicopathological variables. A computer-assisted image analysis of CD3^+^ and CD8^+^ tumor-infiltrating lymphocytes (TIL) density was conducted on surgical specimens of 51 patients with ITAC that underwent a curative treatment including surgery. ITAC displays variable TIL density, which is associated with OS. In a univariate model, the density of CD3^+^ TIL was significantly related to OS (*p* = 0.012), whereas the association with CD8+ TIL density resulted in being non-significant (*p* = 0.056). Patients with intermediate CD3^+^ TIL density were associated with the best outcome, whereas 5-year OS was the lowest for intermediate CD8^+^ TIL density. CD3^+^ TIL density maintained a significant association with OS in the multivariable analysis. TIL density was not significantly related to demographic and clinicopathological variables. CD3^+^ TIL density was independently associated with OS in a non-linear fashion and patients with intermediate CD3^+^ TIL density had the best outcome. Though based on a preliminary analysis on a relatively small series of patients, this finding makes TIL density a potential independent prognostic factor of ITAC.

## 1. Introduction

Intestinal-type adenocarcinoma (ITAC) of the sinonasal tract is a rare malignant tumor with an annual incidence of approximately 1 case per 100,000 inhabitants in Europe [1,2,3]. ITAC has a strong association with occupational exposure to wood, leather, and cork working and resembles colorectal adenocarcinoma from an immunophenotypic and molecular perspective [2,3,4,5]. Despite the advances in imaging, endoscopic surgery, and multimodal therapies [2,3,6,7], ITAC still represents a challenge in view of the potential to spread towards the orbit, skull base, and intracranial space. Among all sinonasal cancers, prognosis settles at an intermediate level, with 5-year overall survival (OS) ranging between 53% and 83% [3].

Several authors, including Barnes and Kleinsasser/Schroeder, have attempted to identify clinically relevant subtypes of this tumor on the basis of histological differences [8,9]. The histologic subtypes have been reported to correlate with differences in clinical behavior, with mucinous and solid subtypes being associated with poorer prognosis [10,11,12]. However, the prognostic value of conventional histopathological parameters in ITACs has been debated [13], and thus novel histopathological variables with potential clinical relevance should be investigated.

Increasing evidence demonstrates that the evolution of cancer is strongly dependent upon the complex interactions within tumor microenvironment (TME), which comprises a variety of entities including fibroblasts, endothelial cells, extracellular matrix, and cells of the immune system. The host immune system is capable of recognizing and eliminating neoplastic cells. Immune cells migrate to tumors and display anti-cancer activity as a result of a tumor antigen-induced immune reaction. However, cancer cells recruit immune regulatory cells and adopt other measures to induce an immunosuppressive network, resulting in the escape from host immunity [14]. For instance, head and neck squamous cell carcinoma can evade immunosurveillance by impairing cancer cell recognition by tumor-infiltrating lymphocytes (TIL) [15]. TIL are mostly represented by T lymphocytes, which is a group of different immune cell types characterized by the expression of the marker CD3. The influence of the type, density, and intratumor location of TIL on patient survival has been reported in colorectal cancer [16,17], which is the first and more comprehensively studied in terms of the prognostic role of TIL, as well as in tumors of the head and neck and several other anatomical sites [18,19,20]. The overall CD8^+^ TIL density measurement alone was further acknowledged to have substantial clinical relevance [21]. Indeed, CD8 marks the subpopulation of T lymphocytes that acquire a specific (i.e., antigen-based) cytotoxic activity against cancer cells. To the best of our knowledge, only one research group has found a prognostic role of CD8^+^ TIL in sinonasal ITAC, independently of tumor stage and histological subtype [22]. The immune TME, with special reference to T lymphocytes and the CD8^+^ cytotoxic subpopulation, is increasingly acquiring relevance in the field of oncology, as understanding the interplay between immune system and cancer is critical to adequately exploit immunotherapy. For instance, the interaction between programmed cell death protein 1 (PD-1), mainly expressed by CD8^+^ lymphocytes, and programmed death-ligand 1 (PD-L1), mainly expressed by cancer cells, was demonstrated as being one of the mechanisms down-regulating immune response against cancer [23]. Indeed, cancers with high expression of PD-L1 have marked immunosuppressive properties and were found to better respond to drugs inhibiting the immune checkpoint interaction between PD-1 and PD-L1. These drugs, such as nivolumab and pembrolizumab, block one of the immunosuppressive strategies of cancer and unleash the cytotoxic action of CD8^+^ lymphocytes [23]. On the other hand, research in the field of immuno-oncology unveiled genetic markers of immunogenicity such as the deficient mismatch repair and inherent microsatellite instability, which are directly related to the tumor mutational burden and thus to the levels of tumor-associated antigens. Understanding whether immune-oncological paradigms learnt from the most frequent cancers such as colorectal adenocarcinoma apply also to rare malignancies is critical to expanding the spectrum of therapeutical options for orphan diseases.

The aim of this retrospective study was to assess the features of immune microenvironment in terms of CD3^+^ and CD8^+^ cells in a series of ITAC and explore their prognostic role, as well as their relations with conventional clinicopathological variables.

## 2. Materials and Methods

### 2.1. Case Selection

All patients with pathological diagnosis of sinonasal ITAC who received a surgery-including curative treatment at the Section of Otorhinolaryngology—Head and Neck Surgery of the University Hospital “Azienda Ospedale Università di Padova” (Padova, Italy) in the timespan 2008–2020 were included. Unavailability of material for histological analysis or insufficient tissue, extensive tumor necrosis, relevant artifacts in available slides, history of immune system disorders, and event-free follow-up shorter than 6 months were considered as exclusion criteria.

Patients were treated and followed according to the principles reported in the “Multi-institutional study on endoscopically treated sinonasal cancers” [12] and the “European position paper on endoscopic management of tumors of the nose, paranasal sinuses, and skull base” [24].

The study was conducted in accordance with the principles of the Helsinki Declaration. All patients signed a detailed informed consent form regarding the processing and publication of their data. They consented to “the use of their clinical data for scientific research purposes in the medical, biomedical and epidemiological fields, also in order to be recalled in the future for follow-up needs”. Data were examined in agreement with the Italian privacy and sensitive data laws, and the internal regulations of the University of Padova.

### 2.2. Staging, Histopathology, Immunohistochemistry, and Image Analysis

For each surgical specimen, all available slides stained with hematoxylin and eosin (H&E) were retrieved from the archives of the Pathology Unit and jointly reviewed by two dedicated pathologists (LA, MS). All cases were re-staged according to the 8th edition of the TNM Classification of Malignant Tumors [25] and classified according to Barnes and Kleinsasser–Schroeder classifications [8,9]. The former one includes five categories: papillary, colonic, solid, mucinous, and mixed subtypes; the latter stratifies ITACs into papillary-tubular cylinder cell type (corresponding to papillary, colonic, and solid subtypes according to Barnes), alveolar goblet cell type and signet-ring cell type (both corresponding to Barnes’ mucinous subtype), and transitional type (corresponding to mixed subtype) [26]. The most representative slide, showing the highest density of immune cells (based on low magnification evaluation under the optical microscope), was identified and the respective formalin-fixed, paraffin-embedded (FFPE) tissue block was used for immunohistochemical analyses.

Serial sections with 2.5-mm-slides were obtained from FFPE blocks and immunohistochemical analysis was performed with an automated system (Benchmark-Ultra, Ventana, Tucson, AZ, USA) using the following antibodies: anti-CD3 (Mouse Monoclonal, clone LN10, 1:100 dilution, Novocastra, Leica Biosystems, Newcastle Upon Tyne, UK) and anti-CD8 (Mouse Monoclonal, clone SN6, dilution 1:100, DakoCytomation, Dako, Glostrup, Denmark).

CD3- and CD8-immunostained slides were scanned (NanoZoomer-SQ C13140-21/NDP.scan for SQ 1.0.8; Hamamatsu Photonics K.K., Hamamatsu, Japan) and the corresponding digital images validated by the abovementioned pathologists. The entire tumor area was manually outlined and considered for the analysis. Image analysis was performed with Visiopharm software, version 4.5.6.5 (Hoersholm, Denmark). CD3^+^ and CD8^+^ TIL were detected in both the intratumoral and stromal compartments. Intratumoral TILs were defined as intraepithelial mononuclear cells within tumor cell nests or in direct contact with tumor cells, and stromal TILs as lymphocytes in the tumor stroma without direct contact with tumor cells [27]. Lacking anatomical orientation and being affected by artifacts due to the type of surgery, available tissue sections were deemed inadequate to analyze separately intratumoral and stromal TIL densities. Thus, cumulative TIL density per mm^2^ including both intratumoral and stromal TIL was measured.

### 2.3. Statistical Analysis

The statistical analysis was performed with R Studio (Vienna, Austria), version 2022.02.3, and X-tile (3.6.1—Yale University, New Haven, CT, USA). Descriptive statistics was used as follows: continuous variables were described through median, mean, standard error, range, and interquartile range, whereas categorical variables through category absolute and percentage distribution. Distribution of continuous variables (age, CD3^+^ TIL density, and CD8^+^ TIL density) was assessed through the Shapiro-Wilk test. Association of CD3^+^ and CD8^+^ TIL density with demographic and clinicopathological information (i.e., age, gender, exposure to wood/leather dust, primary vs. recurrent presentation, presence of nodal metastasis at presentation, ITAC subtype according to Barnes and Kleinsasser–Schroeder classifications, pT category, presence of focal neoplastic necrosis, presence of microscopic lymphovascular invasion, presence of microscopic perineural invasion) was performed with Mann–Whitney U test (dichotomous variables), Kruskal–Wallis test (non-dichotomous categorical variables), or Spearman’s correlation test (continuous variables).

Survival analysis was performed with OS as the primary outcome. Time-to-event data were gathered by measuring the follow-up duration after completion of treatment and classifying the patient’s status at the latest follow-up evaluation as either event-free (i.e., patient alive with or without evidence of disease) or dead of any cause. Disease-specific survival (DSS) and recurrence-free survival (RFS) were considered as secondary outcomes, with events being defined as ITAC-specific death and recurrence, respectively.

Association of CD3^+^ and CD8^+^ cell density with primary and secondary outcomes was analyzed by a univariate Cox proportional-hazards model before and after restricted cubic spline-correction. Restricted cubic spline-correction with 4 degrees of freedom was applied to assess a potential non-linear association between cell density and OS. The best categorization of CD3^+^ and CD8^+^ cell density relative to OS and other outcomes was achieved by defining the optimal cutoffs according to the minimum p- and the maximum χ^2^-values [28]. CD3^+^ and CD8^+^ cell density was then categorized and tested with the log-rank test and in a multivariable OS Cox proportional-hazards model with a priori selection of relevant demographic, clinicopathological, and treatment-related covariates (i.e., age, ITAC subtype according to Barnes classification, pT category, margin status, and adjuvant radiotherapy). Assumptions of the model were checked as follows: proportional hazards assumption was tested with the Schoenfeld test, influential observations with deviance residual analysis, and nonlinearity with Martingale residual analysis. Multi-collinearity of covariates was assessed with a multi-collinearity test; covariates with a variance inflation factor of 4 or higher were considered as multi-collinear.

For all statistical tests, the level of significance was set at 0.05.

## 3. Results

### 3.1. Cohort Description

**Figure 1:**
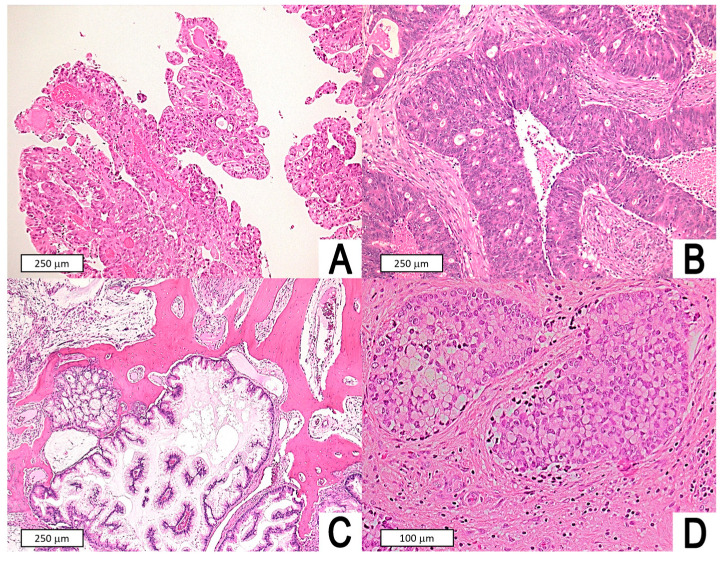
Histomorphological spectrum of intestinal-type adenocarcinoma. (**A**) Papillary subtype (according to Barnes) or papillary-tubular cylinder cell type (according to Kleinsasser and Schroeder) of ITAC displaying prominent papillary fronds with minor amounts of tubular structures; note the resemblance to intestinal villous or tubular adenomas. (**B**) Colonic subtype (according to Barnes) or papillary-tubular cylinder cell type (according to Kleinsasser and Schroeder classification) with solid-cribriform architecture, comedonecrosis, and a striking similarity with large intestine adenocarcinoma. (**C**) Mucinous subtype (according to Barnes) or alveolar goblet cell type (according to Kleinsasser and Schroeder) of ITAC featuring distended glands or cell clusters within pools of extracellular mucin, infiltrating bone tissue. (**D**) Mucinous subtype (according to Barnes) or signet-ring cell type (according to Kleinsasser and Schroeder) of ITAC with solid nests and signet-ring cells. Original magnifications: (**A**–**C**): 100×; (**D**): 200×.

The study included 51 patients, of which 44 (86.3%) were males. Median age at surgery was 70 years (mean: 68; range: 43–88; interquartile range: 60–77). Age was normally distributed (*p* = 0.060). Only 10 (19.6%) patients had no history of exposure to wood or leather dust. Pathological T category was distributed as follows: T1 in 15 (29.4%) cases, T2 in 17 (33.3%), T3 in 3 (5.9%), T4a in 8 (15.7%), and T4b in 8 (15.7%). Only one (2.0%) patient had a clinically appreciable nodal metastasis at presentation. Lymphovascular and perineural invasion were observed in seven (13.7%) and one (2.0%) patients, respectively. Focal neoplastic necrosis was observed in 32 (62.7%) tumors. According to the Barnes classification [8], 26 (51.0%) ITAC were of the colonic type, 11 (21.6%) mucinous, 6 (11.8%) solid, 5 (9.8%) papillary, and 3 (5.9%) mixed. According to the Kleinsasser–Schroeder classification [9], ITAC were classified as papillary-tubular cylinder cell type in 37 (72.5%) cases, alveolar goblet cell type in 8 (15.7%), signet-ring cell type in 4 (7.8%), and transitional type in 2 (3.9%) (Figure 1). Based on definitive histopathological examination, intraoperative findings, and preoperative imaging, the resection was classified as R0 in 34 (66.7%) cases and R1 in the remaining 17 (33.3%), following the principles of multi-block resection [12,29]. Adjuvant radiotherapy was delivered in 35 (68.6%) cases. Eight (15.7%) patients also received cisplatin/carboplatin chemotherapy concomitantly to postoperative radiation therapy.

Mean duration of follow-up was 49.4 months (median 43; standard error: 5; range: 3–132; interquartile range: 13–77). Five-year OS, DSS, and RFS estimates were 62.2%, 78.7%, and 69.6%, respectively.

### 3.2. CD3^+^ and CD8^+^ Tumor-Infiltrating Lymphocytes Density

**Figure 2:**
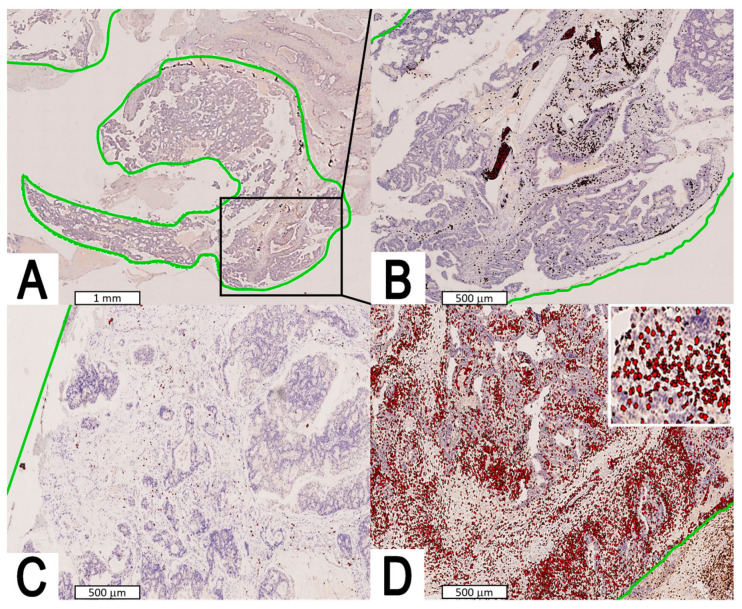
Digital image analysis performed with Visiopharm™ software on immunostained slides. The entire tumor area (green line) was manually selected and considered for the analysis. The density of CD3 TILs per mm^2^ was counted. (**A**) Intermediate CD3^+^ TIL density according to prognostic categorization (see Section 3 and Figure 4 for details). (**B**) Larger view from inset of A. (**C**,**D**) Low and high CD3^+^ TIL density according to prognostic categorization (see Section 3 and Figure 4 for details); the upper-right white square in D shows a magnification of digital cell labelling.

**Figure 3:**
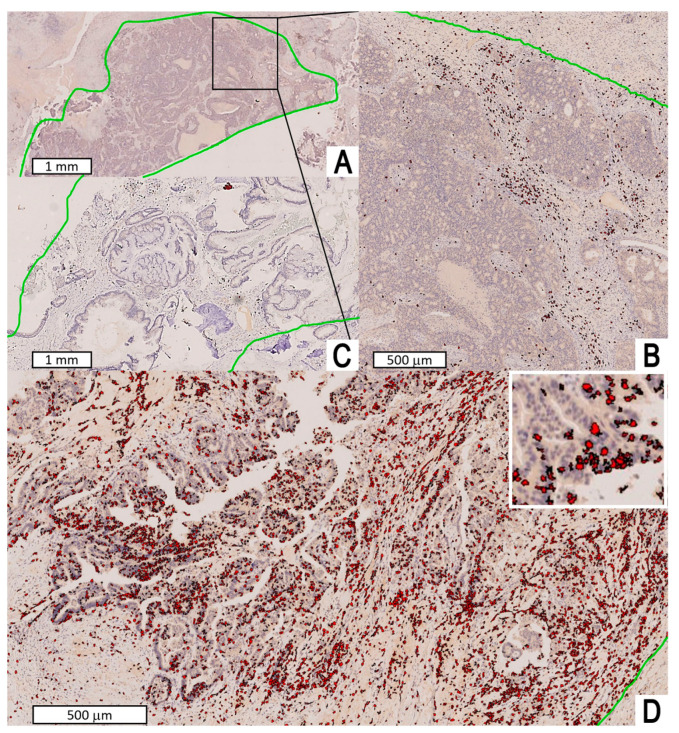
Digital image analysis performed with Visiopharm™ software on immunostained slides. The entire tumor area (green line) was manually selected and considered for the analysis. The density of CD8^+^ TILs per mm^2^ was counted. (**A**) Intermediate CD8^+^ TIL density according to prognostic categorization (see Section 3 and Supplementary Figure S1 for details). (**B**) Larger view from inset of A. (**C**,**D**) Low and high CD8^+^ TIL density according to prognostic categorization (see Section 3 and Supplementary Figure S1 for details); the upper-right white square in D shows a magnification of digital cell labelling.

Median CD3^+^ TIL density was 133.3 cell/mm^2^ (mean: 221.4; standard error: 46.9; range: 17.7–2253.5; interquartile range: 73.4–209.7) (Figure 2). Median CD8^+^ TIL density was 101.3 cell/mm^2^ (mean: 158.5; standard error: 32.9; range: 9.5–1527.9; interquartile range: 54.8–158.7) (Figure 3). The densities of CD3^+^ and CD8^+^ TIL were correlated (r_s_ = 0.715, *p* < 0.0001) and non-normally distributed (both *p* < 0.0001). No significant associations between CD3^+^/CD8^+^ TIL density and clinicopathological features were found (Table 1).

### 3.3. Prognostic Effect of Tumor-Infiltrating Lymphocytes

**Figure 4:**
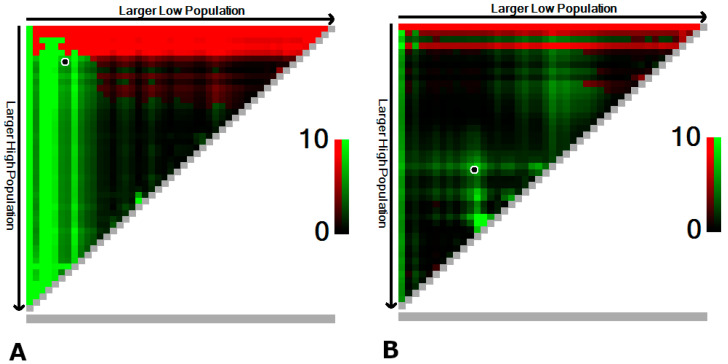
X-tile plots of cut-points for prognostic markers CD3^+^ TIL (**A**) and CD8^+^ TIL (**B**) on a cohort of sinonasal ITAC. Red coloration of cut-points indicates an inverse correlation with OS, whereas green coloration represents a direct association. X-tile discovers optimal cut-points at 45 and 368 cell/mm^2^ for CD3^+^ TIL (**A**), and at 55 and 101 cell/mm^2^ for CD8^+^ TIL (**B**). The optimal cut-points are highlighted by the circles in the panels.

In a univariate model, the density of CD3^+^ TIL was significantly associated with OS (*p* = 0.012), whereas the association with density of CD8^+^ TIL resulted as not significant (*p* = 0.056). When applying the restricted cubic spline correction to the CD3^+^ and CD8^+^ TIL densities, a non-linear association with OS was unveiled. The associations of CD3^+^ and CD8^+^ TIL densities with 5-year OS were inverted-U-shaped and U-shaped, respectively. Given the results of univariate analysis, a three-group categorization was chosen for both CD3^+^ and CD8^+^ TIL densities. The most prognostic cutoff values were 45 and 368 cell/mm^2^ for CD3^+^ TIL and 55 and 101 cell/mm^2^ for CD8^+^ TIL (Figure 4).

**Figure 5:**
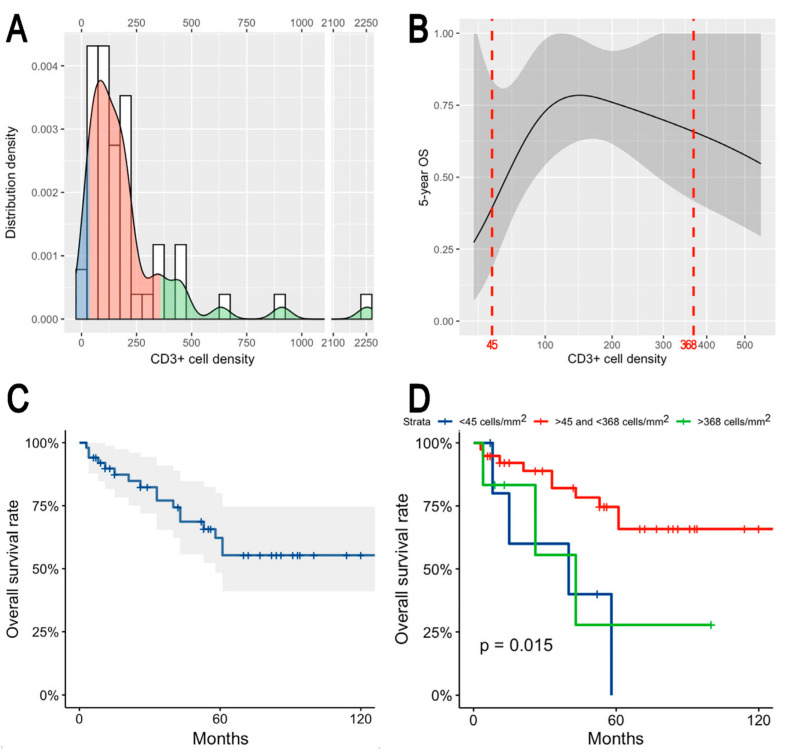
Panel showing continuous and categorized distribution of CD3^+^ cell density (**A**), non-linear association between CD3^+^ cell density and 5-year OS with cutoffs resulting from the X-tile plots reported in (**B**), and Kaplan–Meier plot depicting OS in the whole series (**C**) and according to the 3 resulting classes (**D**).

X-tile-based clustering resulted in the group of patients with intermediate values (n = 39) being associated with higher 5-year OS than those with low (n = 6) or high (n = 6) CD3^+^ TIL density (Figure 5). In contrast, 5-year OS was the lowest for intermediate (n = 12) CD8^+^ TIL density, while it increased for low (n = 14) and high (n = 25) densities (Figure S1). The log-rank test showed that both CD3^+^ and CD8^+^ TIL densities, even categorized, were significantly associated with OS (*p* = 0.015 and *p* = 0.046, respectively) (Figure 5 and Figure S1). When tested in an OS multivariable model including age, subtype according to Barnes classification [8], pT category, margin status, and adjuvant radiotherapy, only the categorized CD3^+^ TIL density (i.e., not the categorized CD8^+^ TIL density) maintained significance as a prognostic factor (Table 2). All the assumptions of the model were checked, and no multicollinearity was found.

In a univariate model, associations of CD3^+^ TIL and CD8^+^ TIL densities with DSS were not significant (*p* = 0.892 and *p* = 0.654, respectively). Similarly, RFS was not significantly associated to the same TIL densities (*p* = 0.949 and *p* = 0.913, respectively). When applying the restricted cubic spline correction to the CD3^+^ TIL density, a non-linear association was observed with DSS and RFS at 5 years (Figure S2). Patients with low CD3^+^ TIL density had a worse prognosis, which resulted in being significant only for DSS (*p* = 0.041 and *p* = 0.190, respectively) (Figure S2). When applying the restricted cubic spline correction to the CD8^+^ TIL density, no association with DSS and RFS was observed.

## 4. Discussion

The present study showed that sinonasal ITAC displays variable TIL density, which is associated with OS. Overall TIL density, which was measured using the T lymphocyte marker CD3, was remarkably variable, ranging from 17.7 to 2253.5 cell/mm^2^. CD8^+^ TIL, which are a subset of CD3^+^ cells with specific cytotoxic activity against cancer cells, were detected in all ITAC, although with a considerable heterogeneity in density (range: 9.5–1527.9 cell/mm^2^). This finding is not in line with the results of a study by García-Marín et al., who reported that roughly one-third (35.3%) of ITAC did not display CD8^+^ cells [22]. The difference could be explained by the methodologies used to evaluate CD8^+^ TIL in the two studies, which was based on computer-assisted quantitative assessment in ours and a semiquantitative approach on tissue microarrays in the study by García-Marín et al. Other reports on immune TME have sometimes shown conflicting results, which could be attributed to the absence of validated and standardized quantification methods. The use of computer-assisted image analysis is time-saving and may enable more robust and repeatable quantification and localization of TIL, TIL subsets, and related biomarker-expressing cells within the TME, with only a few studies applying this tool in head and neck pathology [30,31]. To the best of our knowledge, this is the first study on ITAC evaluating TIL with a computer-assisted image analysis system.

The choice of analyzing CD3^+^ and CD8^+^ TIL was driven by both immunobiological rationale and evidence coming from non-rare cancers immunology. CD3^+^ cells count provides an overall estimate of all TIL populations (i.e., cytotoxic T lymphocytes, T helper cells [both Th1 and Th2], and T regulatory cells), thus expressing whether and to what extent TIL recruitment has occurred. CD8^+^ cells are among the main effectors of specific anti-cancer immunity, and their density is therefore a measure of anti-cancer cytotoxicity occurring in the TME. An immunoscore based on measures of the density of CD3^+^ and CD8^+^ TIL has been validated in a worldwide collaborative study involving more than 4000 patients with solid cancer and showed superior prognostic performance as compared with standard prognosticators in colorectal cancer [32,33].

The crosstalk between ITAC and the immune system is poorly understood. The current available evidence is very limited and comes from two retrospective studies carried out at the University Hospital of Oviedo (Spain) [22,34]. García-Marín et al. analyzed 133 sinonasal ITAC for the presence of CD8^+^ TIL and PD-L1 expression, and showed that: (1) there is a significant association between density in CD8^+^ TIL and expression of PD-L1, with several ITAC characterized by high intratumoral CD8^+^ TIL density displaying also PD-L1 positivity in cancer cells and stromal macrophages; (2) CD8^+^ TIL density was associated with OS irrespective of stage and tumor subtype, with ITAC with high CD8^+^ TIL density being associated with remarkably better prognosis when compared to those with null-to-low CD8^+^ cell infiltrate. They also demonstrated that the TME immune type (classified based on both CD8^+^ TIL density and PD-L1 expression) characterized by high CD8^+^ TIL density was associated with better prognosis irrespective of PD-L1 expression, although with no statistical significance [22]. Riobello et al. reported that the rate of PD-L1 expression in >5% of tumor cells and immune cells was 17% and 33% in ITAC, respectively, as opposed to 34% and 45% in sinonasal squamous cell carcinoma. Moreover, ITAC was found to display focal rather than diffuse expression of PD-L1. At survival analysis, Riobello et al. found that PD-L1-negative ITAC were associated with better disease-free survival when compared with PD-L1-positive ones, but this finding was not confirmed when adjusting by stage and subtype. Overall, these findings led to a hypothesis that ITAC is a poorly immunogenic tumor and that the CD8^+^ TIL density has a more relevant prognostic effect relative to PD-L1 expression. The papillary and colonic subtypes showed more promising immune TME characteristics in the perspective of employing immune checkpoint inhibitors [22]. The present study did not demonstrate a statistically significant association between CD3^+^/CD8^+^ TIL density and demographic and clinicopathological variables. However, papillary ITAC (n = 5) showed a higher density of CD3^+^/CD8^+^ TIL (523.3 vs. 145.8–335.5 cell/mm^2^ and 401.1 vs. 72.8–197.1 cell/mm^2^, respectively), which is consistent with the findings of García-Marín et al. [22], although with no statistical significance. While neither statistically significant nor based on a sufficiently large series, these findings might imply that mechanisms determining the histomorphological phenotype and immune infiltrate are mutually related.

The most interesting finding of the present study is that the CD3^+^/CD8^+^ TIL density was associated with OS in a non-linear fashion. In particular, CD3^+^ TIL density maintained a significant association with OS irrespective of age, histopathological subtype, pT category, margin status, and use of adjuvant radiotherapy, which are all acknowledged prognostic factors (Table 2) [12]. Though limited to this preliminary analysis on a limited series of patients, this finding makes TIL density a potential independent prognostic factor of ITAC with clinical relevance. If confirmed by other studies, this could imply that TIL should be included in the list of factors that drive treatment of sinonasal ITAC, which currently includes histopathological subtype, pT category, and margin status [12,35]. The non-linear association between CD3^+^ TIL density and OS consisted of a cluster of patients with intermediate density (i.e., within the 45–368 cell/mm^2^ range) being associated with improved survival with respect to those with lower or higher values (Figure 5). A similar, inverted-U-shaped association between immune system-related biomarkers, such as the neutrophil-to-lymphocyte ratio, and prognosis was previously described in oral cavity squamous cell carcinoma [36]. On the other hand, a simpler association between populations of the immune contexture and prognosis has been reported in the large majority of studies [19]. However, one should notice that a non-linear approach was rarely used in similar research. Surprisingly, the relationship between CD8^+^ TIL density and OS displayed an opposite trend, with patients with intermediate density values being associated with the worst outcome. However, this association was not statistically significant when adjusting for other relevant prognostic factors at multivariable analysis and included a very narrow cluster of cases with intermediate density (Figure S1). Moreover, CD8^+^ TIL resulted in being not significantly associated with DSS and RFS. Thus, the prognostic effect of CD8^+^ TIL subpopulation on this cohort of patients was eclipsed by other prognosticators and can be considered as less relevant compared with the overall CD3^+^ TIL density measure.

When focusing on the association between CD3^+^ TIL density and OS, some hypotheses on the mechanisms determining the aforesaid non-linear trend can be advanced. Briefly, the low- and high-density clusters could be associated with poorly immunogenic and immune-suppressing/-exhausting ITAC variants, respectively. Tumor immune control is a multistep process in which T lymphocytes play a major role. During the early stages of tumor development, if sufficient immunogenic antigens are produced, naïve T cells are primed in the draining lymph nodes, followed by their concomitant activation and migration to the TME where they recognize specific antigens on cancer cells and eliminate the most immunogenic neoplastic cells [37]. Some cancers can be primarily poorly immunogenic or arise in a subject with a “subclinically” poorly functioning immune system, thus minimizing the recruitment of immune cells and generating a TME with low T cell density. This mechanism could explain the existence of a group of ITAC with low CD3^+^ TIL density and, accordingly, poor prognosis. In cancers with standard immunogenicity, the less immunogenic clones escape the immune control of T cells and survive, a process termed cancer immune editing [37]. In parallel, during tumor development, cancer cells evolve mechanisms that are able to prevent the local cytotoxic response of effector T cells [38]. Accumulating evidence indicates that cancer cells can acquire the ability to suppress immune cell activity and/or induce T cell exhaustion [39]. Importantly, increased levels of exhausted T cells in patients are directly associated with poor prognosis and poor outcomes in various cancers, indicating the importance of modulating the immune TME with immune checkpoint inhibitors [40]. Both cancer-induced immune-suppression and immune-exhaustion mechanisms are reasonably associated with a high density of non-functional T cells and could thus explain the cluster of ITAC with high CD3^+^ TIL density and poor OS. Interestingly, this cluster of patients was associated with decreased OS but equal DSS and RFS with respect to those with intermediate CD3^+^ TIL density (Figure 5, Figures S2 and S3), which might imply that immune-suppression and immune-exhaustion could characterize patients with increased risk of ITAC-unrelated death after completing their cancer treatment. The fact that CD3^+^ but not CD8^+^ TIL density was associated with prognosis raises interest towards the CD3^+^CD8^−^ populations, such as T helper and T regulatory cells. These hypotheses mandate further characterization of lymphocytes and other leucocytes of the ITAC TME to determine whether TIL-poor and TIL-rich tumors display an immunologic profile consistent with the aforesaid mechanisms. Future research of our group will be needed to further clarify the role played by immune cell populations other than CD3^+^ and CD8^+^ cells in the ITAC TME by using markers such as CD4, CD20, CD163, CD68, and CD56.

The main weaknesses of our study are its single-center, retrospective setting as well as the limited number of cases and subsequent limited statistical power. These limitations are inherent to the fact that sinonasal ITAC is a rare cancer and implied the absence of a statistical validation of prognostic findings. Moreover, no usable data on general health conditions of patients could be retrieved, which is a further limitation of the survival analysis. Of note, the surgical expertise of the team alongside with the availability of state-of-the-art technology in our and nearby facilities (e.g., intensity-modulated radiotherapy) improved over the inclusion period, which introduces another potential source of bias. On the other hand, this study was meant to be an exploratory investigation and its main strengths lie in the homogeneity of the series of patients because: (1) all tumors originated in the sinonasal tract and had the same histology; (2) surgical treatment was planned with curative intent and performed consecutively by the same team; (3) all cases were examined by two dedicated head and neck pathologists; (4) only surgical specimens (not biopsies) were considered; (5) both CD3^+^ and CD8^+^ TILs were evaluated; (6) an image-analysis-based approach was utilized; (7) oncological follow-up strategy was standardized.

## 5. Conclusions

Sinonasal ITAC displays variable TIL density, which is associated with OS in a non-linear fashion. TIL density was not significantly associated with demographic and clinicopathological variables. ITACs with intermediate CD3^+^ TIL density were associated with the best outcomes, whereas those with low and high density showed reduced survival. Poor immunogenicity, immune-suppression, and immune-exhaustion are all mechanisms that potentially explain the findings of the present study and mandate to better characterize and dissect the immune-system-ITAC crosstalk in future research.

## Figures and Tables

**Table 1:** Effect of clinical and histopathological variables on the tumor-infiltrating lymphocytes density.

Variable	CD3^+^ Lymphocyte Density (cell/mm^2^)	*p*-Value *	CD8^+^ Lymphocyte Density (cell/mm^2^)	*p*-Value *
Age	r_s_ = −0.127	0.375	r_s_ = −0.101	0.480
Sex	Male: 234.3 Female: 139.7	0.691	Male: 169.1 Female: 91.7	0.460
Exposure to wood and/or leather dust	No: 204.8 Yes: 225.4	0.455	No: 184.1 Yes: 152.3	0.840
Primary or persistent/recurrent tumor	Primary: 217.3 Persistent/recurrent: 269.6	0.362	Primary: 160.7 Persistent/recurrent: 132.9	0.700
Pathologic T stage	T1: 198.0 T2: 217.6 T3: 163.0 T4a: 188.3 T4b: 176.6	0.737	T1: 199.9 T2: 229.4 T3: 183.3 T4a: 205.5 T4b: 172.5	0.544
Nodal status	N0: 222.5 N+: 167.1	0.734	N0: 159.7 N+: 101.3	1.000
Necrosis	Not present: 253.4 Present: 202.3	0.110	Not present: 224.5 Present: 119.3	0.514
Lymphovascular invasion	V0: 193.5 V1: 396.4	0.274	V0: 140.4 V1: 272.1	0.292
Perineural invasion	Pn0: 223.9 Pn1: 94.5	0.497	Pn0: 160.5 Pn1: 58.1	0.541
Histological subtype according to Barnes	Papillary: 523.3 Colonic: 176.1 Solid: 335.5 Mucinous: 145.8 Mixed: 159.5	0.516	Papillary: 401.1 Colonic: 125.7 Solid: 197.1 Mucinous: 128.0 Mixed: 72.8	0.518
Histological subtype according to Kleinsasser and Schroeder	Papillary-tubular cylinder cell: 248:8 Alveolar goblet cell: 145.3 Signet ring-cell:_186.8 Transitional: 86.4	0.465	Papillary-tubular cylinder cell: 174.5 Alveolar goblet cell: 139.6 Signet-ring cell: 86.5 Transitional: 81.7	0.804
Mucinous type	No: 248.8 Yes: 148.7	0.151	No: 174.5 Yes: 116.2	0.375

* Spearman’s rank correlation coefficient for age, Mann–Whitney test for comparisons of 2 groups, Kruskal–Wallis test for comparisons of 3 or more groups.

**Table 2:** Multivariable overall survival models including age, subtype according to Barnes, pathological T category, margin status, adjuvant radiotherapy (RT), and either CD3^+^ or CD8^+^ tumor-infiltrating lymphocytes density.

Covariate	CD3^+^ TIL Density-Including Model		CD8^+^ TIL Density-Including Model	
	HR (95%-CI)	*p*-Value	HR (95%-CI)	*p*-Value
Categorized CD3^+^ TIL density	<45 cell/mm^2^: REF 45–368 cell/mm^2^: 0.23 (0.06–0.98) >368 cell/mm^2^: 0.50 (0.08–3.28)	REF **0.046** 0.468	Not included	
Categorized CD8^+^ TIL density	Not included		<55 cell/mm^2^: REF 55–101 cell/mm^2^: 0.98 (0.17–5.52) >101 cell/mm^2^: 0.38 (0.10–1.50)	REF 0.982 0.167
Age	1.03 (0.96–1.09)	0.398	1.05 (0.98–1.12)	0.212
ITAC subtype according to Barnes	Papillary, colonic, mixed: REF Solid, mucinous: 0.29 (0.05–1.74)	REF 0.174	Papillary, colonic, mixed: REF Solid, mucinous: 0.65 (0.10–4.20)	REF 0.655
Pathological T category	T1-3: REF T4: 3.20 (0.83–12.34)	0.091	T1-3: REF T4: 1.63 (0.34–7.76)	REF 0.538
Margin status	Uninvolved: REF Involved: 1.58 (0.47–5.35)	0.464	Uninvolved: REF Involved: 2.06 (0.51–8.33)	REF 0.310
Adjuvant RT	No: REF Yes: 0.17 (0.05–0.56)	REF **0.004**	No: REF Yes: 0.15 (0.04–0.51)	REF **0.002**

Significant *p*-values are reported with bold style.

## Data Availability

The data presented in this study are available on request from the corresponding author.

## Supplementary Materials

The following supporting information can be downloaded at: https://www.mdpi.com/article/10.3390/jpm13050726/s1, Figure S1. Panel showing continuous and categorized distribution of CD8^+^ cell density (A), non-linear association between CD8^+^ cell density and 5-year OS with cutoffs resulting from the X-tile plots reported in Figure 5B, and Kaplan–Meier plot depicting OS in the whole series (C) and according to the three resulting classes (D); Figure S2. Panel showing non-linear association between CD8^+^ cell density and 5-year DSS and RFS with cutoffs resulting from the X-tile analysis; Figure S3. Cumulative incidence plot depicting occurrence of cancer-specific and cancer-unrelated deaths in patients clustered in three classes as per CD3^+^ cell density (A: <45 cell/mm^2^; B: >45 and <368 cell/mm^2^; C: >368 cell/mm^2^).
