# Supplementary figures and images for: The Prognostic Role of the Immune Microenvironment in Sinonasal Intestinal-Type Adenocarcinoma: A Computer-Assisted Image Analysis of CD3+ and CD8+ Tumor-Infiltrating Lymphocytes
Source: J Pers Med. 2023 Apr 25;13(5):726. doi: 10.3390/jpm13050726 (PMC10219337; doi:10.3390/jpm13050726)

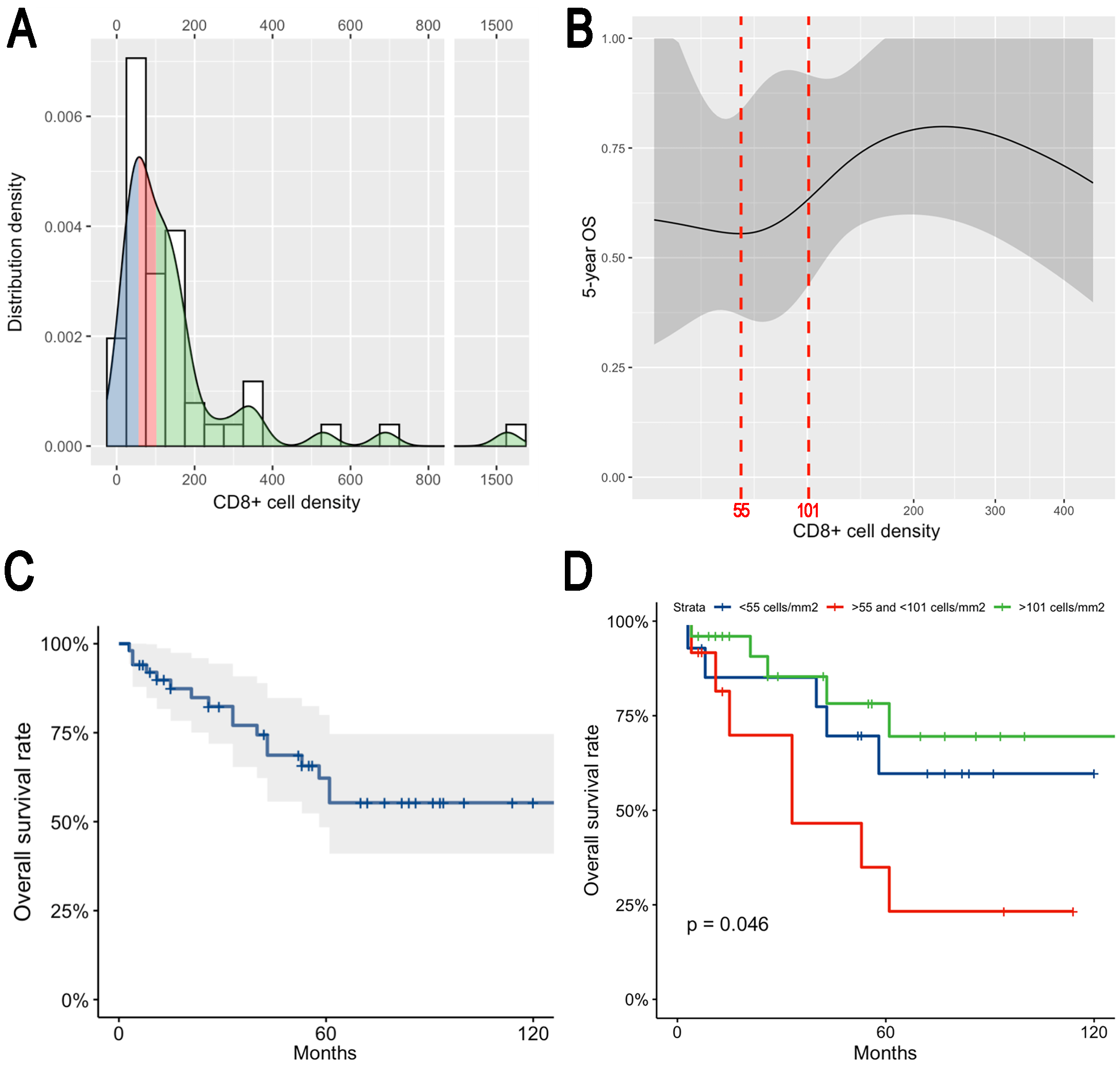

Supplement: Supplementary file 1 [file jpm-13-00726-s001.zip › FIGURE_S1.tif]

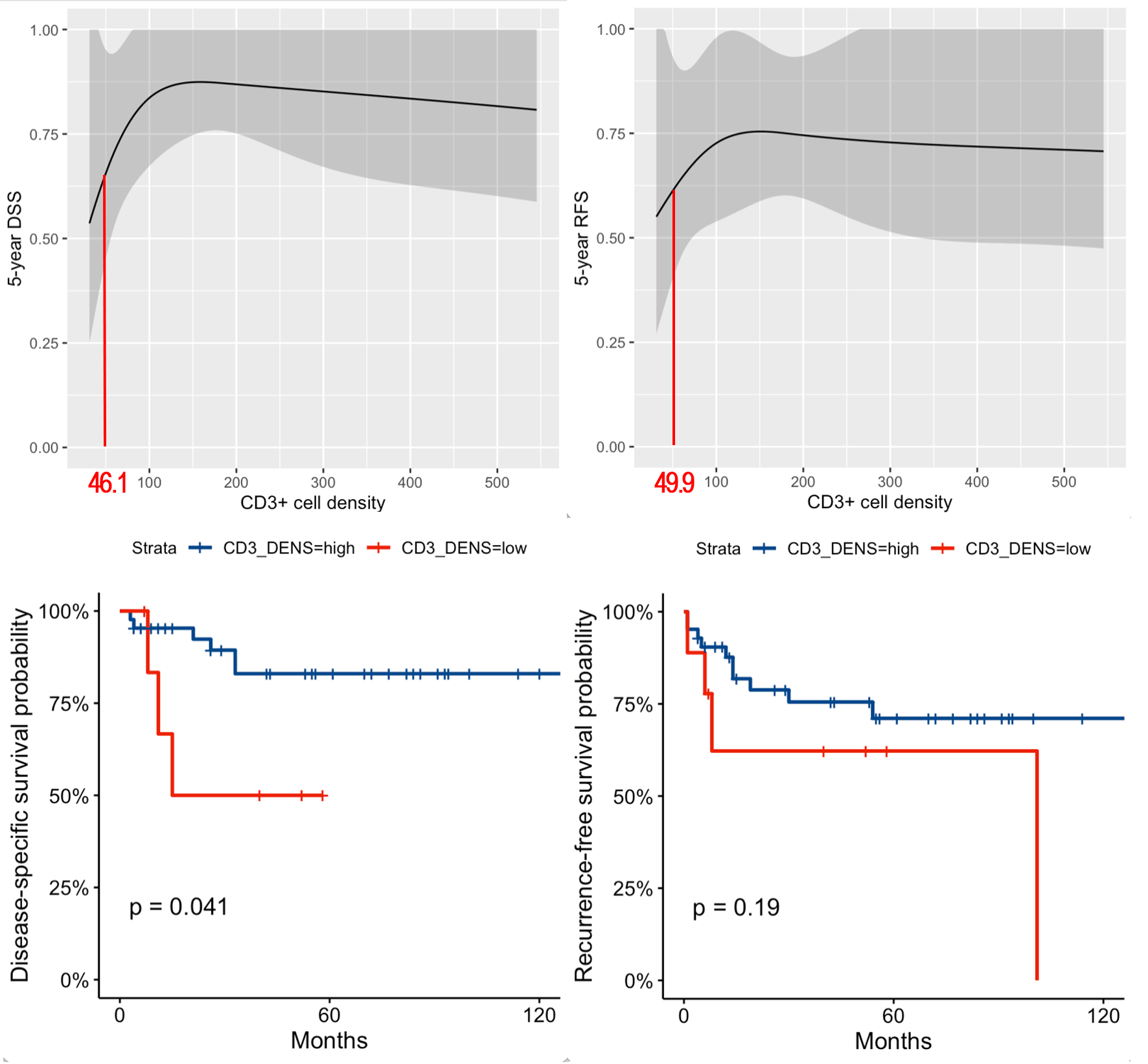

Supplement: Supplementary file 1 [file jpm-13-00726-s001.zip › FIGURE_S2.tif]

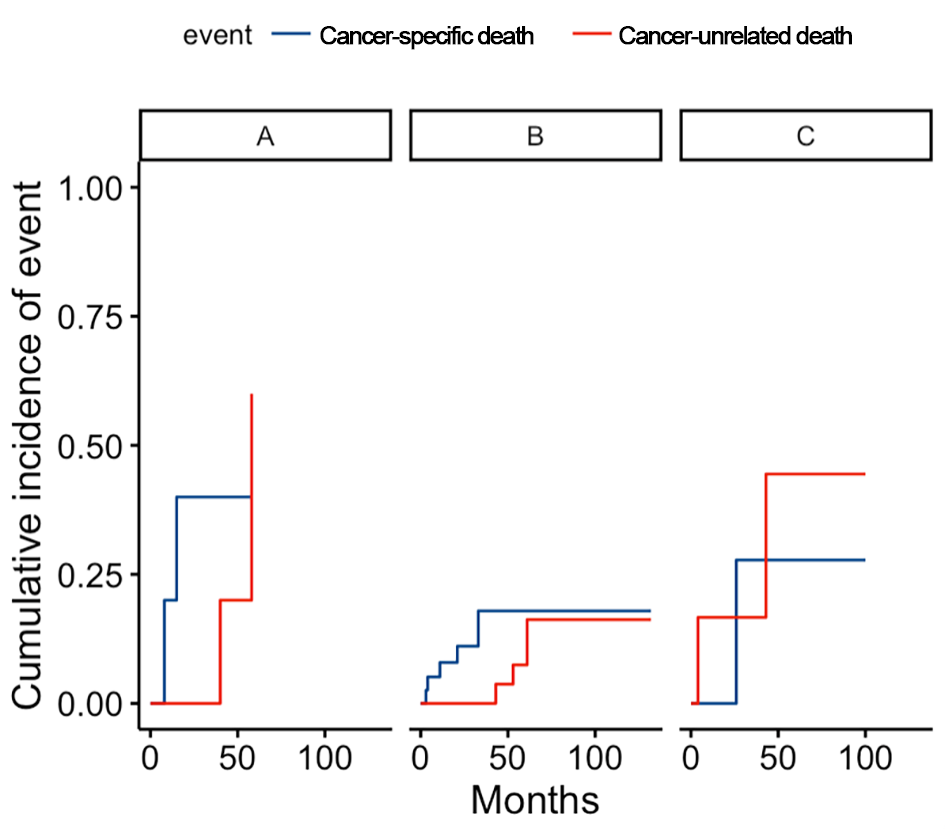

Supplement: Supplementary file 1 [file jpm-13-00726-s001.zip › FIGURE_S3.tif]
